# AMH Regulation by Steroids in the Mammalian Testis: Underlying Mechanisms and Clinical Implications

**DOI:** 10.3389/fendo.2022.906381

**Published:** 2022-05-31

**Authors:** Nadia Y. Edelsztein, Clara Valeri, María M. Lovaisa, Helena F. Schteingart, Rodolfo A. Rey

**Affiliations:** ^1^ Centro de Investigaciones Endocrinológicas “Dr. César Bergadá” (CEDIE), CONICET – FEI – División de Endocrinología, Hospital de Niños Ricardo Gutiérrez, Buenos Aires, Argentina; ^2^ Departamento de Biología Celular, Histología, Embriología y Genética, Facultad de Medicina, Universidad de Buenos Aires, Buenos Aires, Argentina

**Keywords:** androgen insensitivity, androgen response element, estradiol, estrogen response element, dihydrotestosterone, ketoconazole, testotoxicosis, triptorelin

## Abstract

Anti-Müllerian hormone (AMH) is a distinctive biomarker of the immature Sertoli cell. AMH expression, triggered by specific transcription factors upon fetal Sertoli cells differentiation independently of gonadotropins or sex steroids, drives Müllerian duct regression in the male, preventing the development of the uterus and Fallopian tubes. AMH continues to be highly expressed by Sertoli until the onset of puberty, when it is downregulated to low adult levels. FSH increases testicular AMH output by promoting immature Sertoli cell proliferation and individual cell expression. AMH secretion also showcases a differential regulation exerted by intratesticular levels of androgens and estrogens. In the fetus and the newborn, Sertoli cells do not express the androgen receptor, and the high androgen concentrations do not affect AMH expression. Conversely, estrogens can stimulate AMH production because estrogen receptors are present in Sertoli cells and aromatase is stimulated by FSH. During childhood, sex steroids levels are very low and do not play a physiological role on AMH production. However, hyperestrogenic states upregulate AMH expression. During puberty, testosterone inhibition of AMH expression overrides stimulation by estrogens and FSH. The direct effects of sex steroids on AMH transcription are mediated by androgen receptor and estrogen receptor α action on *AMH* promoter sequences. A modest estrogen action is also mediated by the membrane G-coupled estrogen receptor GPER. The understanding of these complex regulatory mechanisms helps in the interpretation of serum AMH levels found in physiological or pathological conditions, which underscores the importance of serum AMH as a biomarker of intratesticular steroid concentrations.

## 1 Introduction

Anti-Müllerian hormone (AMH) is a glycoprotein hormone (1) that belongs to the transforming growth factor beta (TGFβ) superfamily (2). It is produced essentially by Sertoli cells of the testis and granulosa cells of the ovary: after castration, AMH is no longer detected in serum (3, 4). AMH is synthesized as a homodimeric precursor consisting of two identical polypeptide chains, with a large N-terminal pro-region of 110-kDa and a small C-terminal mature domain of 25-kDa. AMH is subjected to post-translational proteolytic processing (5); the resulting N-terminal and C-terminal dimers remain associated in a non-covalent complex that is biologically active (6, 7). The pro-region is displaced from the non-covalent complex upon binding to an AMH receptor homodimer (8). AMH transduces its signal through a specific type II receptor, AMHR2, that is expressed at the cell surface in target organs (9) and interacts with the non-specific type I receptors ACVR1 (initially called ALK2), BMPR1A (ALK3) or BMPR1B (ALK6) (reviewed in (10). The most widely recognized action of AMH takes place during male fetal development, where it provokes the regression of the Müllerian ducts –the anlagen of the Fallopian tubes, the uterus and the upper part of the vagina (reviewed in (11).

**Figure 1:**
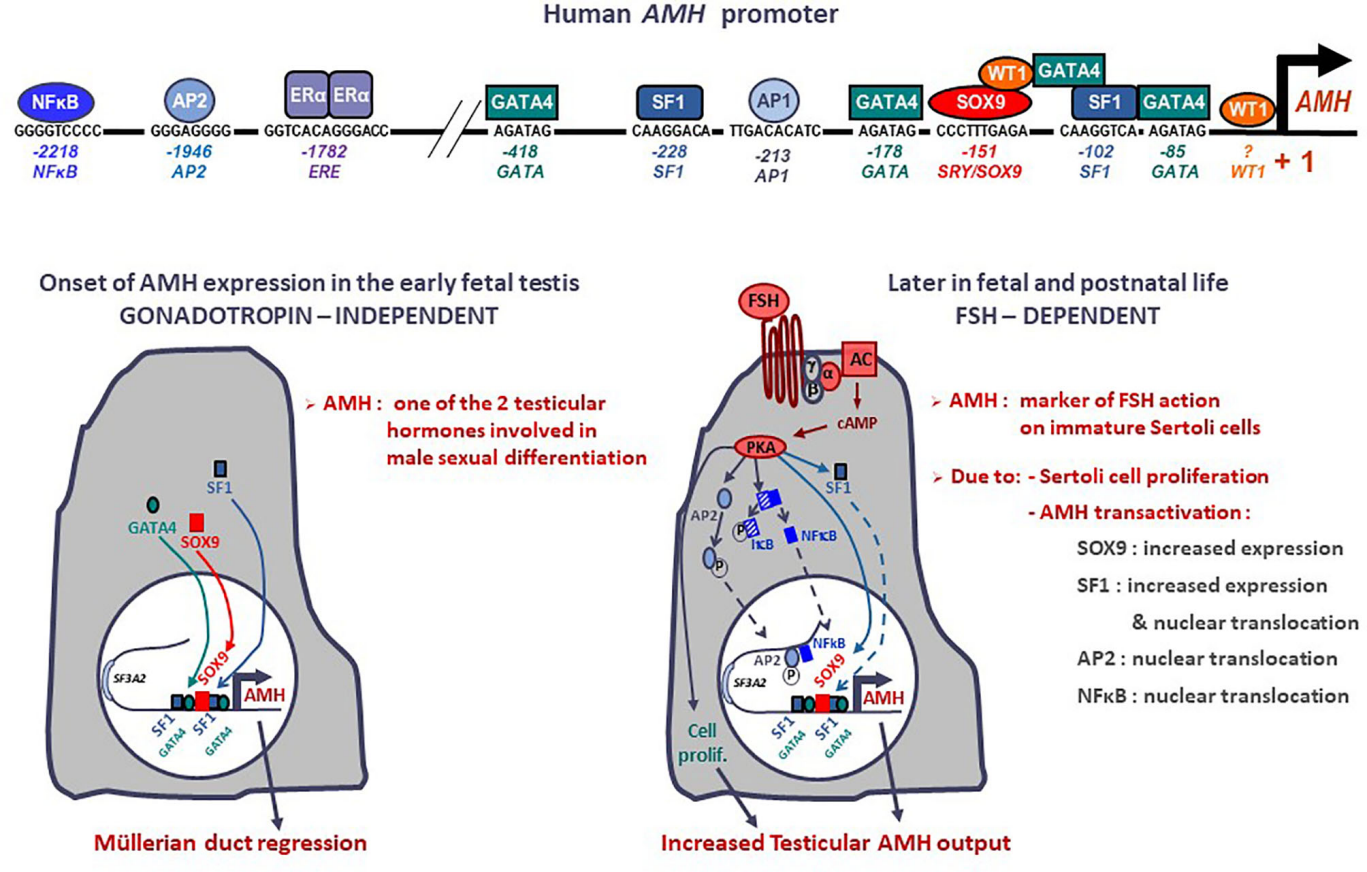
Onset and upregulation of *AMH* expression. Transcription factors SOX9, SF1, GATA4 and WT1 bind to specific response elements in the proximal *AMH* promoter and trigger *AMH* expression in early fetal life, independently of gonadotropin or steroid action, resulting in Müllerian duct regression. From the second trimester of gestation, FSH increases testicular AMH production by 2 different mechanisms: it promotes cell proliferation and upregulates *AMH* transcription *via* the transduction pathway involving the heterotrimeric (α/β/γ) G protein, adenylyl cyclase (AC), cyclic AMP and protein kinase A (PKA) resulting in increased expression and nuclear translocation of transcription factors SOX9, SF1, AP2 and NFκB. Modified with permission from Grinspon and Rey (14), ^©^ 2010 S. Karger AG.

The human *AMH* gene is located on chromosome 19p13.3 (12) and consists of 5 exons spanning 2.8 kb (2). The 3’ end of the 5^th^ exon encodes the biologically active C-terminal domain of the protein. In the testis, the major transcription initiation site is located 10 bp upstream of the ATG codon (13). Most of the best characterized binding sites for transactivating factors with a relevant role in fetal life lie within the proximal 500 bp (**Figure 1**) (reviewed in (15), but other response elements for factors involved in AMH regulation in postnatal life are present in the distal promoter (16). Indeed, distal promoter sequences are necessary for the maintenance of AMH expression in the testis during postnatal life (17). While no canonical androgen response elements (AREs) have been described (18), a consensus sequence for estrogen receptor binding (ERE) is present at -1782 bp in the human *AMH* promoter (13, 19).

### 1.1 The Ontogeny of AMH Expression in the Testis

**Figure 2:**
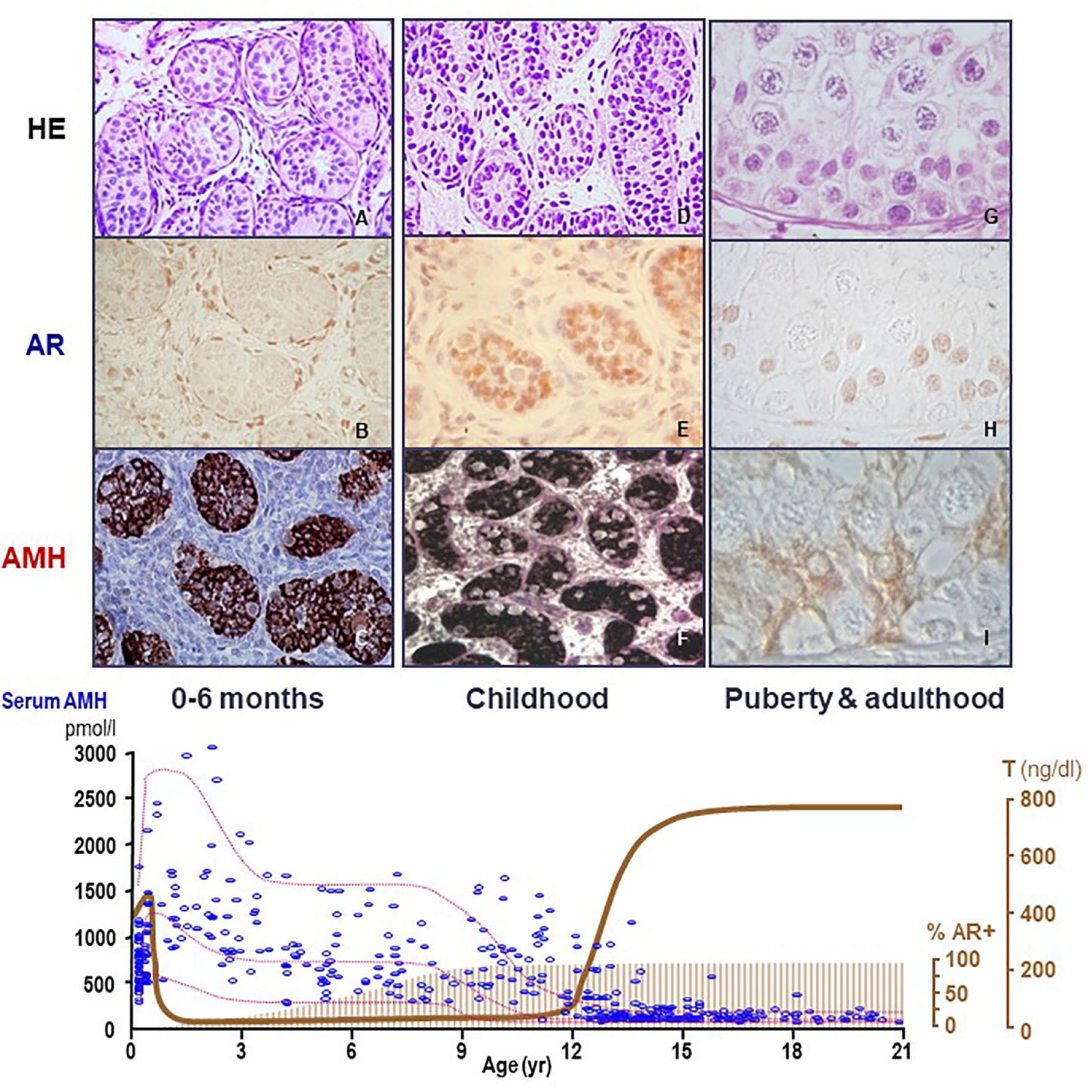
Relationship between AMH production, androgen receptor (AR) expression and androgen levels in the human testis. During the postnatal period of activation of the hypothalamic-pituitary-testicular axis (0-6 months, first column, **A–C**), testosterone (T) levels are high (bottom graph, serum hormone levels); however, Sertoli cells do not show maturational changes because they do not express the AR (upper graph, second line, immunohistochemistry). Therefore, AMH expression is high (upper graph, third line, AMH immunohistochemistry) and germ cells do not enter meiosis (upper graph, first line, HE: hematoxylin-eosin staining). During the “quiescent” period of the gonadal axis (childhood, second column, **D–F**), androgen synthesis is negligible, which explains why Sertoli cells remain immature even though they have started to express the AR. In puberty and adulthood (third column, **G–I**), T increases, inducing Sertoli cell maturation, reflected in the decline of AMH expression and the onset of adult spermatogenesis. Bottom graph shows % AR+: percentage of Sertoli cells expressing the AR. AMH (pmol/l) and T (ng/dl): schematic representation of AMH and T serum levels from birth to adulthood in males.

**Figure 3:**
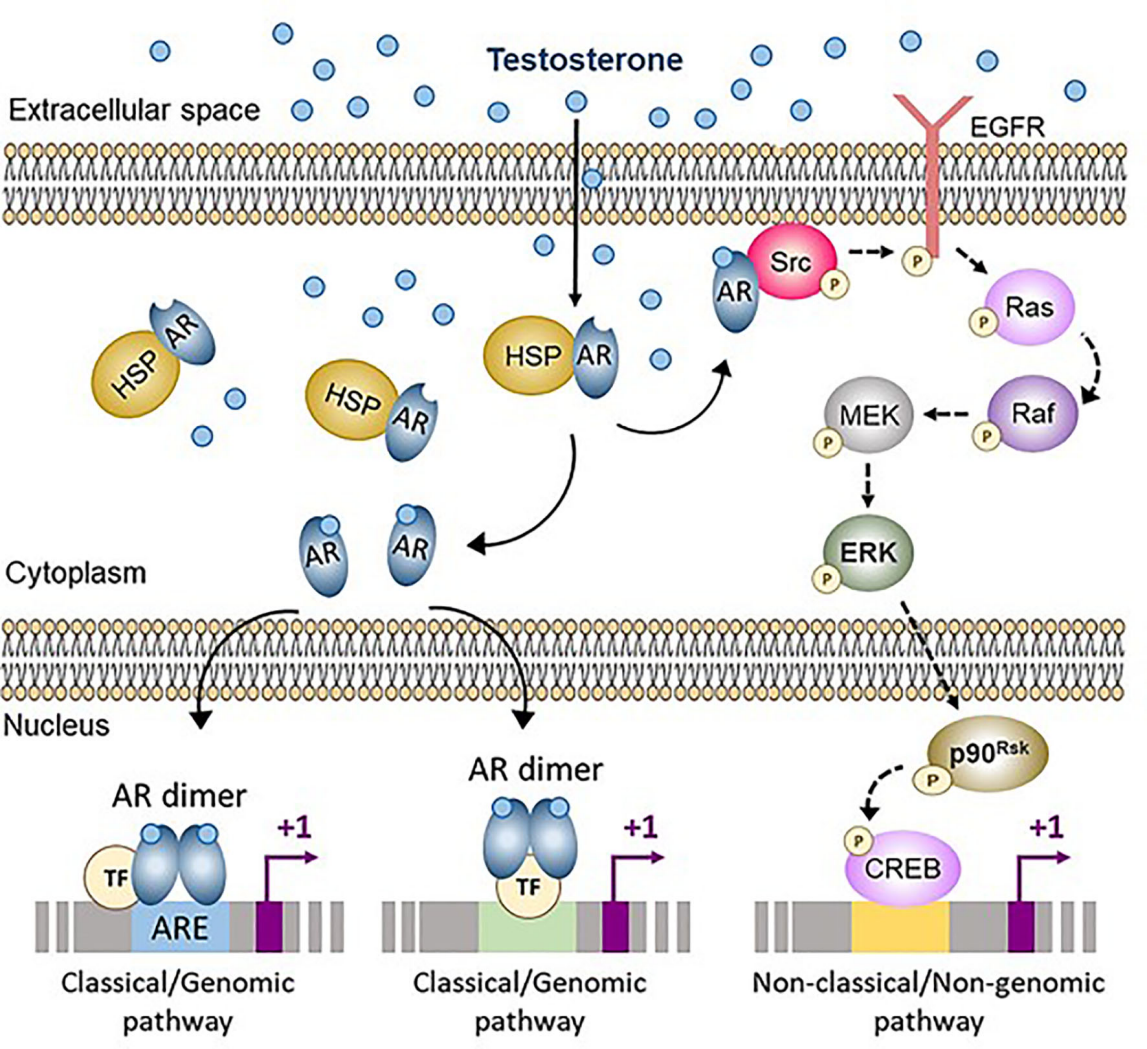
Classical (genomic) and non-classical (non-genomic) androgen signaling in Sertoli cells. In cells not exposed to androgens, the androgen receptor (AR) is bound to heat-shock proteins (HSP) in the cytoplasm. Testosterone and other androgens are steroids that easily cross the cell membrane and bind to the AR, that is released from HSP. The ligand-bound AR can either translocate to the nucleus and interact with androgen response elements (ARE) or with other transcription factors (TF), thus activating the classical/genomic pathway, or migrate to the inner side of the cell membrane and interact with Src, thus activating the non-classical/non-genomic pathway. CREB, cAMP response element binding protein; EGFR, Epidermal growth factor receptor; ERK, Extracellular signal-regulated kinase; MEK, Mitogen-activated protein kinase; Src, Steroid receptor coactivator. Reproduced with permission from Edelsztein and Rey (18) ^©^ 2019 The Authors.

AMH is one of the earliest cell-specific proteins produced by Sertoli cells when the testis differentiates from the gonadal ridge in the XY embryo (20). AMH starts to be expressed at 12.5 days post-coitum in the mouse (21) and from the 8^th^ week onwards in the human (22). Although its role in sex differentiation of the internal ducts takes place in the first phases of fetal development (see below), AMH continues to be produced by Sertoli cells at very high levels during the whole fetal life and, postnatally, until the onset of puberty (**Figure 2**). During pubertal development, Sertoli cells progressively express less AMH (25), and the directional secretion switches from the basal to the adluminal compartment (26). Consequently, AMH concentration in serum is high during childhood but decreases during puberty, when it increases in seminal plasma [reviewed in ref (27)]. The decrease in AMH expression coincides with the establishment of the blood-testis barrier and the onset of germ cell meiosis (**Figure 3**) (28–30), which are androgen-dependent processes. During adulthood, serum AMH is approximately ten- to twentyfold lower as compared to the prepubertal period in males (24), but still twofold higher than in females (31).

### 1.2 Physiological Role of AMH in Male Development

In mammals, the earliest stages of intrauterine development are characterized by the existence of a sexual undifferentiated period where no differences can be observed, except at the chromosomal level, between XX and XY embryos. The gonadal ridges and the precursors of the external genitalia are identical in both sexes, and there are two sets of internal ducts: Wolffian ducts that give rise to the male internal genitalia, and the Müllerian ducts that differentiate into the female internal genitalia. The pioneering work by Alfred Jost (32) demonstrated the existence of a Mullerian inhibiting activity, specific to the fetal testis, explaining why the female internal organs do not develop in the male fetus. In the absence of AMH action, e.g. in individuals with mutations of the genes coding for AMH or its specific receptor AMHR2, the Fallopian tubes and the uterus are present [reviewed in ref (15)]. The absence of AMH action is also evidenced in 46,XY disorders of sex development (DSD) due to complete gonadal dysgenesis [reviewed in ref (33)]. Interestingly, the window of action of AMH on Müllerian ducts is limited to the earliest stages of intrauterine life (34); this explains why the development of internal genitalia is not disrupted by ovarian AMH production that starts when Müllerian ducts are already insensitive to its action (35). The fact that AMH continues to be secreted by the testis after the critical window of Müllerian duct regression and that it is produced by the ovaries at clearly detectable levels suggest that AMH may have other physiological roles. Nonetheless, individuals with *AMH* or *AMHR2* null mutations resulting in complete loss of AMH expression or action do not show other evident phenotypes [reviewed in ref (11)].

### 1.3 AMH as a Biomarker in Physiological and Pathological States

Independently of its physiological role(s), AMH is a proven biomarker of the immature Sertoli cell population in normal and pathological conditions [reviewed in ref (36–39)]. Serum AMH reflects the mass of immature Sertoli cells, i.e. of functional testicular tissue, from fetal life to adulthood. In the fetus (22), newborn and child (40), detectable AMH in serum indicates the existence of testes, which is particularly useful for the differential diagnosis between bilateral cryptorchidism and anorchidism in boys with nonpalpable gonads. In patients with DSD, serum AMH levels are commensurate with the amount of testicular tissue, except for those with a Persistent Müllerian duct syndrome due to *AMH* mutations (41). Undetectable AMH is indicative of no testicular development in 46,XY girls with complete gonadal dysgenesis. Circulating levels are below the normal male range in patients with 46,XY DSD due to partial gonadal dysgenesis –where both Sertoli and Leydig cell development is impaired– but normal or high in 46,XY DSD due to isolated steroidogenic defects –where only Leydig cell function is affected– or to androgen insensitivity or 5α-reductase deficiency –where both Leydig and Sertoli cell development is preserved (33). In patients with 46,XX DSD, serum AMH above the female range indicates the existence of functional testicular tissue suggesting the diagnosis of ovotesticular or testicular DSD, in contrast with other forms of XX virilization, such as aromatase deficiency, congenital adrenal hyperplasia of androgen-secreting tumors, which present with serum AMH in the female range (42). In normally virilized boys, low serum AMH is seen in patients with primary (24, 43, 44) or central hypogonadism (45). Conversely, high AMH is suggestive of Sertoli cell tumors (46), excessive signaling downstream the FSH receptor pathway like in McCune-Albright syndrome (47), or hyperestrogenism (19). During pubertal development, the decline in serum AMH levels is an early sign of Sertoli cell maturation (48), while an excessive decrease to undetectable levels is typical of Klinefelter syndrome (49). Conversely, the persistence of elevated AMH levels despite an increase in testosterone production is indicative of androgen insensitivity (50).

## 2 Steroid-Independent Regulation of Testicular AMH Expression

### 2.1 AMH Expression During Early Fetal Development

The onset of AMH expression in the fetal testis is independent of gonadotrophin or steroid regulation (**Figure 1**). SOX9, upregulated by SRY, triggers *AMH* expression by binding to a specific element on the proximal *AMH* promoter (51). Subsequently SF1 (51–53), GATA4 (54) and WT1 (55) cooperate to further upregulate *AMH* transcription whereas DAX1 represses the transcriptional cooperation between SF1 and GATA4 (56).

As mentioned, although its action takes place in the early stages of fetal sex differentiation, AMH continues to be produced by Sertoli cells throughout life (**Figure 2**). The proximal *AMH* promoter plays a major role in the initiation of fetal expression, when AMH induces the regression of Müllerian ducts, but it proves insufficient to maintain AMH expression thereafter, when sequences farthest from the transcription start site are required (17).

### 2.2 AMH Regulation by FSH in Late Fetal and Postnatal Life

From the second half of gestation, FSH increases testicular AMH output through two different mechanisms (**Figure 1**): it induces Sertoli cell proliferation and upregulates AMH expression at the individual cell level (16, 30). The continuous effect of high FSH levels on Sertoli cells for almost one year (the last 6 months of fetal life plus the 3-6 months of postnatal life induces a progressive increase in serum AMH (57), which peaks at 5-6 months of age (58). This explains why serum AMH levels are low, in coincidence with small testicular size, in boys with congenital central (hypogonadotrophic) hypogonadism (45, 59) and increase after FSH treatment (60, 61). The underlying molecular mechanisms include the classical cyclic AMP-dependent pathway triggered by the FSH receptor coupled to the Gαs protein (16), which activates SOX9 (62) and SF1 (62) acting on the proximal *AMH* promoter, but also NFκB and AP2, which bind to response elements lying approximately 2 kb upstream of the *AMH* start site (62). These molecular mechanisms explain the elevated AMH production observed in boys with McCune-Albright syndrome carrying a gain-of-function somatic mutation in the *GNAS1* gene encoding the Gαs protein (47, 63).

### 2.3 Lack of Androgen-Mediated Regulation of Testicular AMH Expression in Fetal and Early Postnatal Life

The fetal period and the stage of postnatal activation of the hypothalamic-pituitary-testicular axis occurring during the first 3 to 6 months after birth, usually referred to as “mini-puberty”, are characterized by the coexistence of high testosterone levels and AMH expression in the testes (**Figure 2**). The continuous exposure to high androgen levels for approximately one year neither affects AMH expression nor induces histologic maturational changes in Sertoli cells or spermatogenic onset. Immunohistochemical studies have clarified why: Sertoli cells do not express the AR and, therefore, they are physiologically insensitive to androgens until the end of the first year of life in humans (**Figure 2**). Subsequently during infancy and childhood, AR expression increases progressively in Sertoli cells until full expression is observed by the age of approximately 8 years (64–66). An equivalent ontogeny can be observed in rodents (30, 67). Interestingly, boys with high intratesticular androgen concentration due to precocious puberty show normal serum AMH during the first year of life but declining levels thereafter, when the AR starts to be expressed in Sertoli cells (68).

## 3 Regulation of Testicular AMH Production By Androgens

### 3.1 Downregulation of Testicular AMH Expression at Puberty

Testosterone is a well-known inducer of Sertoli cell maturation during puberty (69). During childhood, the pituitary-gonadal axis is mostly quiescent: LH levels are very low or undetectable and typical Leydig cells are absent in the testis interstitial tissue. Therefore, although Sertoli cells already express the androgen receptor (AR), they are not exposed to androgens (**Figure 2**). When the axis reactivates at the beginning of pubertal development, LH induces the differentiation of Leydig cells that start producing testosterone. Androgen concentration increases rapidly within the testis, even though this is not reflected in the circulating levels until more advanced pubertal stages. Intratesticular testosterone triggers a number of morphologic and functional changes in Sertoli cells [reviewed in ref (18)]. One distinctive change is the decrease in AMH production that occurs as one of the first clinical signs of pubertal development in boys (48), as well as in numerous other mammals (3, 25, 70, 71), in coincidence with the onset of germ cell meiosis (50). Results obtained in experimental mouse models support these observations (30). In the naturally occurring *Tfm* mice, carrying a functionally impaired AR (30), and in mice with an artificially mutated *Ar* gene (72), AMH expression does not wain at the expected age of puberty.

#### 3.1.1 Molecular Mechanisms

Androgen signaling in target cells typically occurs through the AR engaging either the classical (or genomic) or the non-classical (or non-genomic) pathways (**Figure 3**) (73). The classical pathway involves the intracellular AR acting as a transcription factor. In the inactive state, the AR is bound to cytoplasmic heat-shock proteins. When testosterone or the more potent and non-aromatizable androgen dihydrotestosterone (DHT) binds to the AR, a conformational change leads to the release of the AR from the heat-shock proteins, AR phosphorylation, homodimer formation, nuclear translocation and interaction with androgen response elements (ARE), specific DNA sequences in the regulatory regions of target genes (18). Typical ARE are palindromic: in humans, the consensus sequence is 5’-AGAACAnnnTGTTCT-3’ (74). The androgen-bound AR can also regulate target gene expression in the absence of ARE, by interacting with trans-activating factors that bind to their specific binding sequences (75). Androgen signaling through the genomic pathway requires 30-45 minutes to induce transcriptional regulation (76).

Non-classical pathways induce cellular changes more rapidly, within seconds to minutes (**Figure 3**). Androgen action leads the AR to localize near the plasma membrane, where it induces Src. This tyrosine kinase phosphorylates the epidermal growth factor receptor, which triggers the MAP kinase cascade ultimately resulting in the phosphorylation of specific transcription factors (73). Alternatively, the Zn^2+^ transporter ZIP9 has been proposed as a membrane AR that induces the phosphorylation of Erk1/2 and the transcription factors CREB and ATF1 (77).

**Figure 4:**
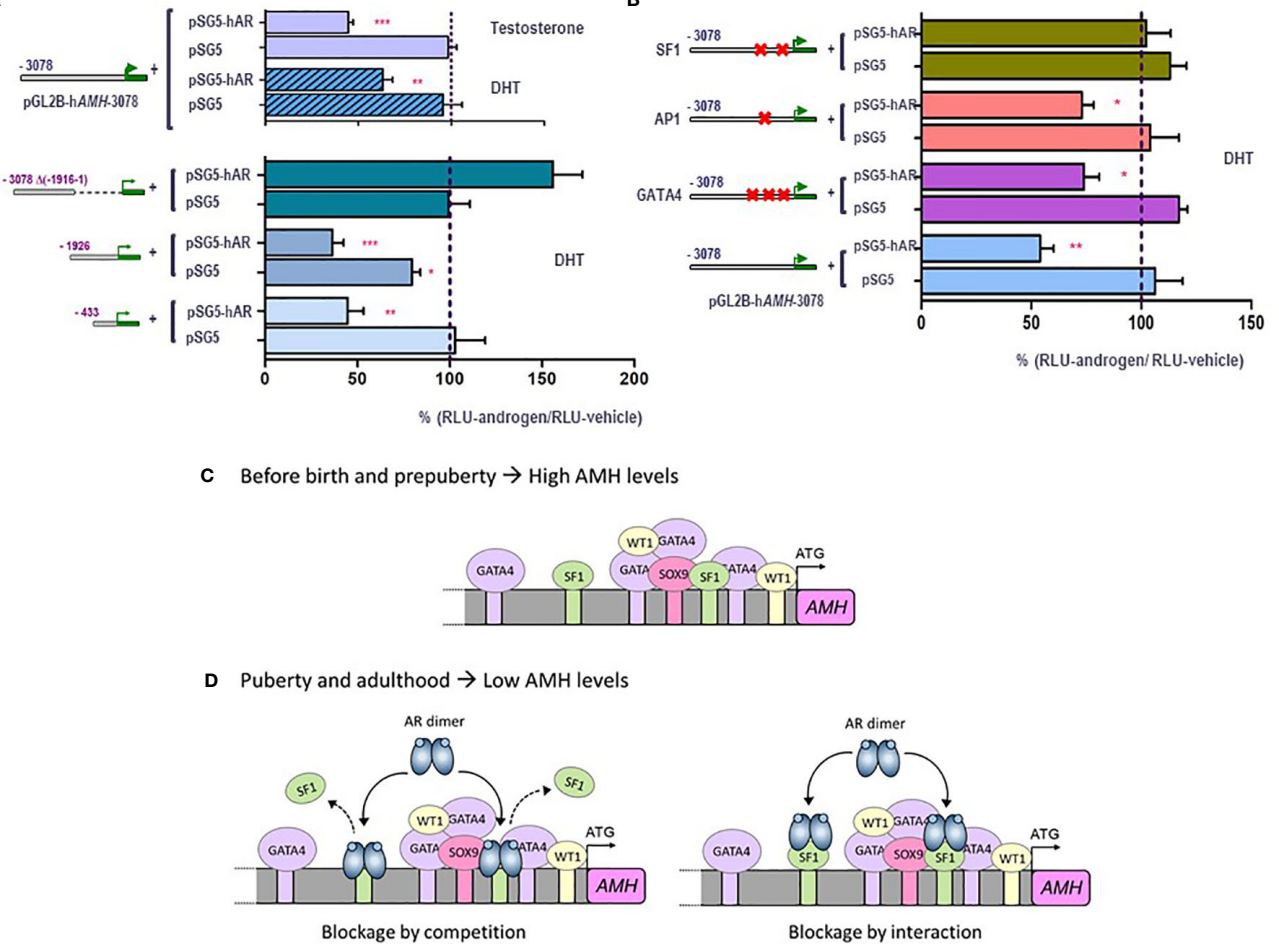
Molecular mechanisms involved in androgen-induced downregulation of *AMH* expression in Sertoli cells. A and B: luciferase assays in SMAT1 Sertoli cells transfected with a luciferase plasmid under control of the human *AMH* promoter (pGL2B-hAMH) of different lengths and co-transfected with an expression plasmid of the androgen receptor (pSG5-AR) or pSG5 devoid of the AR. Cells were exposed to testosterone or DHT 10^-7^ M, or vehicle, and results were expressed as relative luciferase units (RLU) comparing cells exposed to androgens and those exposed to vehicle (* p<0.05, ** p<0.01, *** p<0.001). A 100% level indicates the basal *AMH* promoter activity. Testosterone and DHT induce an inhibition of *AMH* promoter activity when the AR is present but not in its absence, when 433 to 3076 bp of the promoter are present but not when the proximal 1916-bp sequences are lacking **(A)**. The inhibition induced by androgen persists even if the binding sites for AP1 or GATA4 are mutated, but not when SF1 sites are mutated **(B)**. **(C, D)** schematic of androgen regulation of AMH expression. In fetal and postnatal periods before the onset of puberty, the lack of androgen action allows the *AMH* promoter activity induced by SOX9, SF1 and GATA4, resulting in high AMH production. During puberty and adulthood, the steroid-bound AR represses *AMH* promoter activity by competition or by interaction with SF1. Modified with permission from Edelsztein et al. (80) ^©^ 2018 The Authors and Edelsztein and Rey (18) ^©^ 2019 The Authors.

Traditionally, maturation changes induced by androgens were believed to result from upregulation of target genes. However, high-throughput techniques have revealed similar amounts of up-regulated and down-regulated genes in response to androgen action in the maturing testis (78). However, the pathways underlying androgen-mediated downregulation have been barely explored. Using the mouse prepubertal Sertoli cell line SMAT1 (79), we have recently clarified the molecular mechanisms involved in AMH downregulation during puberty (80). The decrease in AMH production is a direct effect of androgens on Sertoli cells, not involving negative feedback on pituitary gonadotrophins or down-regulation of activating transcription factors. Indeed, FSH and testosterone have opposing effects on AMH expression at puberty, with the inhibitory effect of androgens largely exceeding the stimulatory effect of FSH. In SMAT1 cells, both testosterone and the nonaromatizable androgen DHT decrease the activity of a 3-kb human *AMH* promoter in the presence, but not in the absence, of the AR (**Figures 4A, B**), and this inhibition is prevented by the antiandrogen bicalutamide. Using human *AMH* promoters of different lengths in luciferase assays, we demonstrated that androgen-mediated downregulation involved the promoter sequences lying within the proximal 430 bp (**Figure 4A**). No canonical ARE can be found in the *AMH* promoter, and intact SF1 response elements are required for the negative regulation by androgens to occur (**Figure 4B**). This inhibitory effect on *AMH* expression could be mediated by a direct interaction between the ligand-bound AR and the SF1 elements, i.e. blockage by competition, or alternatively due to a protein-protein interaction between the ligand-bound AR and promoter-bound SF1, i.e. blockage by interaction. In any case, the AR prevents SF1 from upregulating *AMH* promoter activity (**Figures 4C, D**).

#### 3.1.2 Clinical Implications

**Figure 5:**
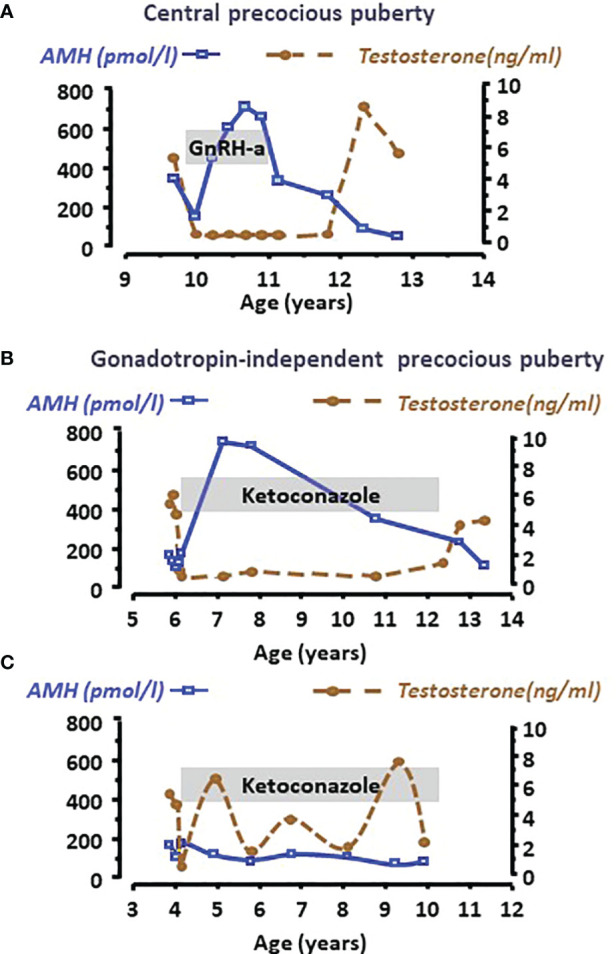
Serum AMH as a biomarker of intratesticular androgen concentration in boys with central precocious puberty **(A)** or with gonadotropin-independent precocious puberty **(B, C)**. Serum AMH is low at diagnosis, indicating the inhibitory effect of high androgen concentration reflected in high serum testosterone levels in all cases. When testosterone production is effectively curtailed by the adequate treatment, such as a GnRH analogue in the patient with central precocious puberty **(A)** or ketoconazole in the patient with gonadotropin-independent precocious puberty **(B)**, Sertoli cells recover their immature status and increase AMH production until treatment is discontinued. Conversely, when adherence to treatment is erratic **(C)**, intratesticular and serum testosterone concentration does not remain at prepubertal levels and Sertoli cells do not revert their pubertal status, which is reflected in low serum AMH. Modified with permission from Rey et al. (48) ^©^ 1993 The Endocrine Society.

Clinical studies have shown that AMH decline is not merely associated with chronological age but rather with the maturation status of the testes. Indeed, a decrease in serum AMH is an early sign of an increase in intratesticular testosterone concentration reflecting the activation of LH pulses occurring with pubertal onset. The decrease in serum AMH occurs already in Tanner 2 stage of puberty in boys, that is earlier than the increase in serum testosterone, taking place in Tanner 3 stage (24, 48, 81, 82). Furthermore, AMH is low for age in boys aged 2 to 8 years with precocious puberty, independently of gonadotrophin levels, and recovers prepubertal levels when testosterone production is effectively curtailed by treatment (**Figures 5A, B**) (48, 68). Serum AMH can be used as a marker of effective treatment. In fact, for Sertoli cells to resume their prepubertal status and increase AMH production, intratesticular androgen levels should remain continuously low for at least 6 months. If adherence to treatment is erratic and intratesticular testosterone concentration shows intermittent variations, serum AMH never increases to prepubertal levels (**Figure 5C**) (48).

On the other hand, testicular AMH production remains high in adolescents and adults with androgen insensitivity due to mutations in the *AR* gene (50, 83–85). The increase in AMH expression has been ascribed to SOX9 upregulation in Sertoli cells (86).

## 4 Regulation of Testicular AMH Production By Estrogens

Like for androgens, the testis is both a source and a target organ of estrogens, synthesized from androgens by the cytochrome P450 enzyme aromatase encoded *CYP19A1*. Aromatase expression is present in the postnatal testis (64) and is stimulated by FSH action on Sertoli cells (87). Estrogen signaling involves 3 different receptors. The canonical nuclear estrogen receptors α (ERα) and β (ERβ) act through their binding capacity on DNA sequences known as estrogen response elements (ERE). ERα and ERβ show a 97% identity in their DNA-binding domains, while their ligand-binding domains have an identity of 60%. Both ERs bind to estrogens with a similar affinity (88). The membrane ER, named GPR30, GPER1 or simply GPER, is a seven-transmembrane domain, G protein-coupled receptor, predominantly present in the endoplasmic reticulum, that mediates rapid cellular responses involving second messengers, ion channels and kinase activities (89).

The 3 ERs are expressed in the testis, but there are differences in the timing and cellular types according to species (90). Sertoli cells of the prepubertal and pubertal human testis express ERα (19) and ERβ (19, 64). In rodents, ERα expression predominates in the prepubertal testis while ERβ is more abundant in the adult (91). GPER has been identified in Sertoli cells from the onset of puberty (92).

### 4.1 Upregulation of Testicular AMH Expression in Postnatal Life

At the onset of puberty, testicular AMH expression increases concomitantly with FSH and estradiol (E2) levels in normal boys (93). This simultaneous elevation in the 3 hormones is also observed in patients with androgen insensitivity, in whom the inhibitory effect of androgens on AMH is disrupted. Patients with androgen insensitivity show increased aromatization of androgens to estrogens, resulting in the development of breasts. As already discussed, AMH upregulation can be explained by a direct action of FSH on Sertoli cell proliferation and on AMH transcription. However, AMH is also elevated in boys with Peutz-Jeghers syndrome (46), in whom Sertoli cell proliferations produce high estrogen levels leading to suppressed FSH (46). Interestingly, the human *AMH* promoter contains a half ERE (**Figure 1**) (13), and E2 regulates AMH expression in the ovary (94).

**Figure 6:**
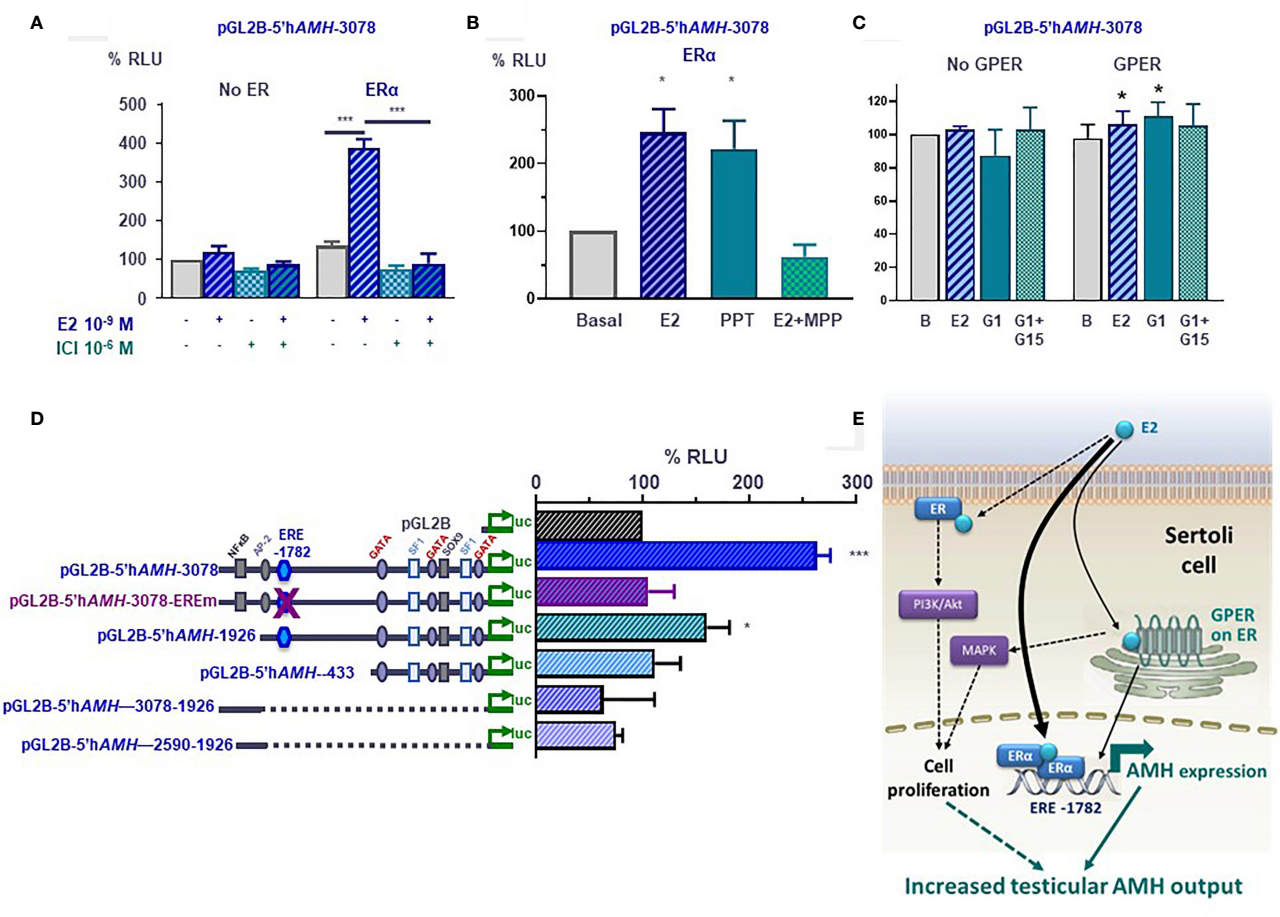
Molecular mechanisms involved in estrogen-induced upregulation of *AMH* expression in Sertoli cells. **(A–D)** Luciferase assays in SMAT1 Sertoli cells transfected with a luciferase plasmid under control of the human *AMH* promoter (pGL2B-hAMH) of different lengths and co-transfected with an expression plasmid of the estrogen receptor alfa (ERα) or the G protein-coupled estrogen receptor (GPER) or the control plasmids devoid of the estrogen receptor sequences. Cells were exposed to estradiol (E2), the ERα/β antagonist ICI 182780, the ERα-specific agonist PPT or antagonist MPP, or the GPER-specific agonist G1 or antagonist G15. Results were expressed as relative luciferase units (RLU) comparing cells exposed to estrogens, agonists and/or antagonists (* p<0.05, *** p<0.001). A 100% level indicates the basal *AMH* promoter activity. E2 induce an upregulation of *AMH* promoter activity when the ERα or the GPER is present but not in their absence **(A–C)**, and when the estrogen response element (ERE) at -1782 of the promoter is conserved **(D)**. **(E)** Proposed model for E2 regulation of *AMH* expression in Sertoli cells. E2 upregulates AMH transcription *via* ERα binding to the ERE. GPER also upregulates *AMH* expression more modestly. The increased AMH expression results in a higher testicular AMH production. Another potential mechanism is the increase in Sertoli cell proliferation induced by membrane-bound ERα signaling through the PI3K/Akt pathway, and/or GPER *via* MAPK signaling. Reproduced with permission from Valeri et al. (19)^©^ 2020 The Authors.

We have recently tested the hypothesis that E2 upregulates AMH expression in the prepubertal and pubertal testis, using experimental mouse models. Treatment of 4-day-old mice with ICI 182780, an antagonist of ERα and ERβ, resulted in significant decrease in serum AMH (**Figure 6A**), indicating that abolishing ER signaling results in a decreased testicular AMH production. Nuclear ERs (91) and GPER (95) are involved in Sertoli cell proliferation, which could in part explain the increase in AMH output by the testes exposed to high estrogen levels.

#### 4.1.1 Molecular Mechanisms

The existence of an ERE on the *AMH* promoter prompted us to test whether estrogens could have a direct effect on AMH expression at the individual Sertoli cell level. Luciferase assays clearly indicated that the activity of a 3-kb human *AMH* promoter was increased after exposure to E2 of SMAT1 Sertoli cells transfected with any of the 3 ERs. However, to elicit a significant response ERβ-transfected SMAT1 needed to be incubated with E2 10^-7^ M (19), a concentration 100-fold higher than that observed in the testes of adolescents (96), while ERα- and GPER-transfected SMAT1 cells showed a maximal response to E2 at the physiological concentration of 10^-9^ M (19). These observations suggest that ERα and GPER, but not ERβ, are involved in the upregulation of AMH expression by E2. Further support was provided by experiments showing that PPT, a potent and selective ERα agonist, increased *AMH* promoter activity while MPP, a specific ERα antagonist, inhibited the *AMH* promoter activity (**Figure 6B**). The effect induced by E2 *via* GPER was more modest. Its specificity was confirmed when SMAT1 cells were exposed to the potent and selective GPER agonist G1 or to the selective GPER antagonist G-15 (**Figure 6C**).

The upregulation induced by E2 in SMAT1 cells on the 3-kb *AMH* promoter was also observed when a 1926-bp promoter with an intact ERE present at position -1782 was transfected, but no estrogenic activity was detected when using *AMH* promoter constructs consisting of the proximal 433 bp or of sequences lying between -1926 and -2590 or between -1926 and -3078, which are devoid of the half-ERE (**Figure 6D**) (19). Furthermore, the response to E2 was abolished by a mutation introduced in the half-ERE at -1782 by site-directed mutagenesis. A direct interaction between ERα and the half-ERE was confirmed in electro mobility shift assays. Complex formation with ERα was observed when ERE wild-type probes, but not with mutant ERE probes, were used. Altogether, these results indicate that E2 at physiological concentrations increases the activity of the *AMH* promoter through ERα binding to the half-ERE site present at position -1782 (**Figure 6E**). The intracellular pathway involved in the more modest *AMH* promoter upregulation induced through the GPER still needs to be elucidated.

#### 4.1.2 Clinical Implications

The cellular and molecular mechanisms involving ERα and, more modestly, GPER in E2 induction of the activity of the *AMH* promoter (19) and of Sertoli cell proliferation (91) provide biological plausibility to explain the increase in serum AMH levels observed in patients with androgen insensitivity (50, 84) or with Peutz-Jeghers syndrome (46). It could also explain why serum AMH is low despite elevated circulating E2 in estrogen insensitivity caused by defective ERα function (97).

## 5 Concluding Remarks

**Figure 7:**
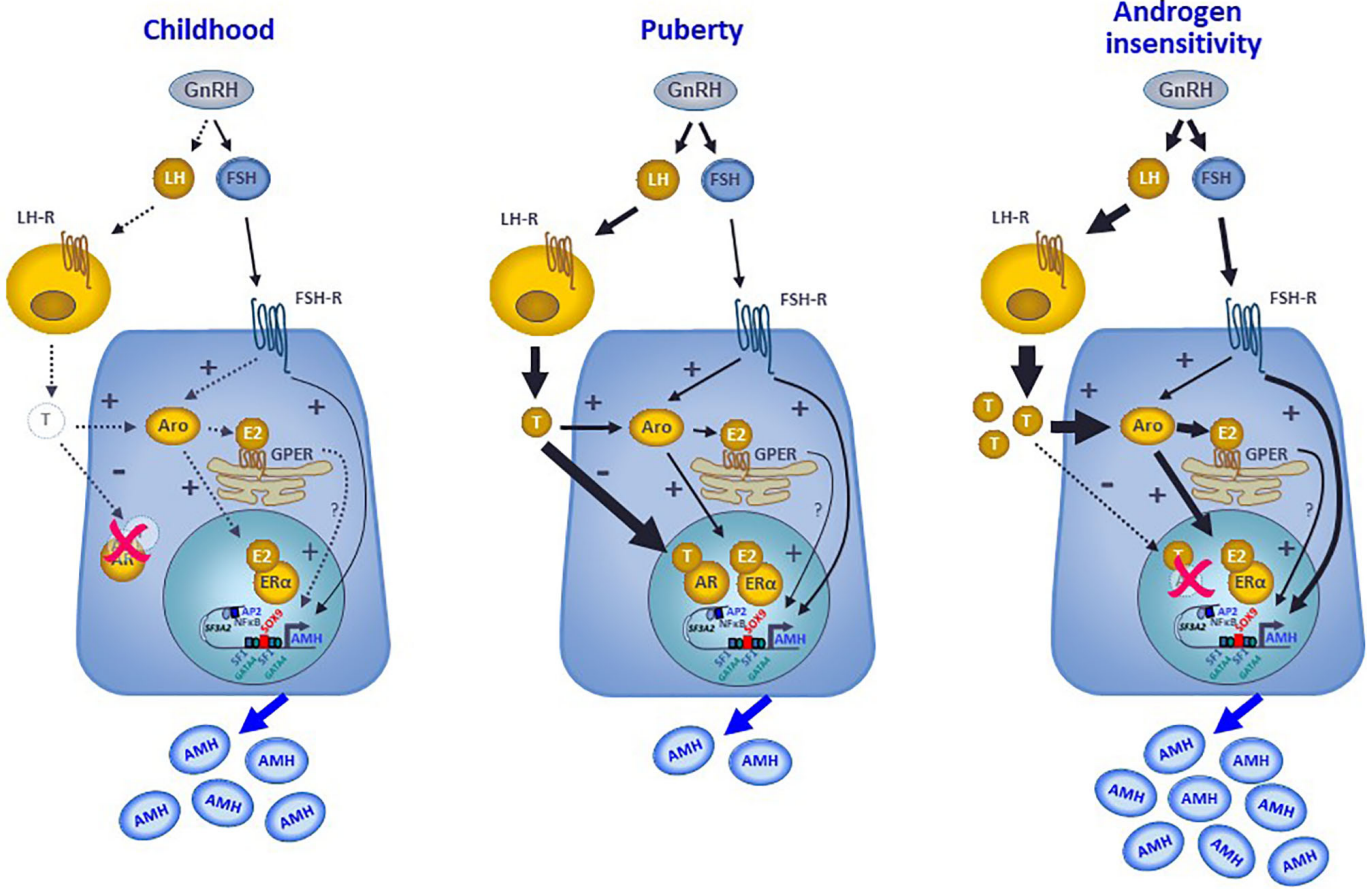
Interaction between androgens, estrogens and FSH on the regulation of AMH expression in Sertoli cells. During childhood, the hypothalamic-pituitary-gonadal axis is quiescent, and the extremely low steroid levels do not exert any physiological regulation on AMH production that is mostly hormone-independent. At puberty, the reactivation of the GnRH neuron and the gonadotropes result in higher LH and FSH levels. LH induces testosterone secretion by testicular Leydig cells. FSH acts on its receptor in the Sertoli cell membrane, resulting in a direct upregulation of AMH expression, through the cyclic AMP-PKA pathway involving transcription factors SOX9, SF1, AP2 and NFκB, and in an indirect upregulation of AMH by inducing aromatase expression. Aromatase converts androgens into estrogens, which can upregulate AMH directly by binding to the nuclear ERα or indirectly acting through the GPER expressed in the membrane of the endoplasmic reticulum. Nonetheless, the inhibitory effect of androgens overrides the stimulatory effect of FSH and estrogens on AMH expression, resulting in a decreased AMH secretion. In hyperestrogenic states with abrogated androgen action, such as the androgen insensitivity syndrome, the inhibitory effect of androgens does not exist, FSH and LH increase resulting in high testosterone that is converted to estradiol. Consequently, AMH production is substantially boosted.

AMH production by Sertoli cells reflects the differential regulation exerted by intratesticular levels of androgens and estrogens in the prepubertal testis, with specific variations according to the developmental stage. In the fetus and the newborn, the high intratesticular androgen concentrations do not regulate AMH expression because the AR is not yet expressed in Sertoli cells, whereas intratesticular estradiol can moderately increase AMH production since ERs are present in Sertoli cells and aromatase activity resulting in androgen conversion to estrogens is stimulated by FSH. During childhood in humans, or the quiescent period of the hypothalamic-pituitary-gonadal axis described in most mammals, the extremely low steroid levels do not exert any physiological regulation on AMH production (**Figure 7**). However, precocious androgen synthesis results in AMH downregulation since the AR is expressed in Sertoli cells from the second year of life. On the contrary, testis-borne hyperestrogenic states provoke an increase in AMH expression (**Figure 7**). Finally, during puberty the inhibitory effect of intratesticular testosterone levels overcome the stimulatory effects of estrogens and FSH (**Figure 7**). Apart from the indirect regulation of testicular function that they exert through their feedback on gonadotropins, both androgens and estrogens have a direct effect on *AMH* gene transcription. These direct effects are mediated by the classical mechanism involving nuclear AR and ER receptor activity on target gene promoters and, more modestly, by estrogen action through the membrane GPER (**Figure 7**). The comprehension of these complex regulatory mechanisms helps in the interpretation of serum AMH levels found in physiological or pathological conditions, highlighting the capacity of serum AMH as a biomarker of intratesticular steroid concentrations, which are not always reflected in serum levels.

## Author Contributions

All authors listed have made a substantial, direct and intellectual contribution to the work, and approved it for publication.

## Conflict of Interest

Until 2020, RR received royalties for the development of an AMH ELISA kit and honoraria for technology services using the AMH ELISA.

The remaining authors declare that the research was conducted in the absence of any commercial or financial relationships that could be construed as a potential conflict of interest.

## Publisher’s Note

All claims expressed in this article are solely those of the authors and do not necessarily represent those of their affiliated organizations, or those of the publisher, the editors and the reviewers. Any product that may be evaluated in this article, or claim that may be made by its manufacturer, is not guaranteed or endorsed by the publisher.

## References

[1] Picard JY Tran D Josso N. Biosynthesis of Labelled Anti-Müllerian Hormone by Fetal Testes: Evidence for the Glycoprotein Nature of the Hormone and for Its Disulfide-Bonded Structure. Mol Cell Endocrinology (1978) 12:17–30. doi: 10.1016/0303-7207(78)90098-9 720747

[2] Cate RL Mattaliano RJ Hession C Tizard R Farber NM Cheung A. Isolation of the Bovine and Human Genes for Müllerian Inhibiting Substance and Expression of the Human Gene in Animal Cells. Cell (1986) 45:685–98. doi: 10.1016/0092-8674(86)90783-x 3754790

[3] Vigier B Tran D du Mesnil du Buisson F Heyman Y Josso N. Use of Monoclonal Antibody Techniques to Study the Ontogeny of Bovine Anti-Müllerian Hormone. J Reprod Fertil (1983) 69:207–14. doi: 10.1530/jrf.0.0690207 6350571

[4] Rey RA Lhommé C Marcillac I Lahlou N Duvillard P Josso N. Antimüllerian Hormone as a Serum Marker of Granulosa Cell Tumors of the Ovary: Comparative Study With Serum Alpha-Inhibin and Estradiol. Am J Obstet Gynecol (1996) 174:958–65. doi: 10.1016/s0002-9378(96)70333-2 8633676

[5] Pepinsky RB Sinclair LK Chow EP Mattaliano RJ Manganaro TF Donahoe PK. Proteolytic Processing of Mullerian Inhibiting Substance Produces a Transforming Growth Factor-Beta-Like Fragment. J Biol Chem (1988) 263:18961–4. doi: 10.1016/S0021-9258(18)37375-7 2974034

[6] Wilson CA di Clemente N Ehrenfels C Pepinsky RB Josso N Vigier B. Müllerian Inhibiting Substance Requires its N-Terminal Domain for Maintenance of Biological Activity, a Novel Finding Within the Transforming Growth Factor-Beta Superfamily. Mol Endocrinol (1993) 7:247–57. doi: 10.1210/mend.7.2.8469238 8469238

[7] Nachtigal MW Ingraham HA. Bioactivation of Mullerian Inhibiting Substance During Gonadal Development by a Kex2/Subtilisin-Like Endoprotease. Proc Natl Acad Sci United States America (1996) 93:7711–6. doi: 10.1073/pnas.93.15.7711 PMC38812 8755541

[8] Cate RL di Clemente N Racine C Groome NP Pepinsky RB Whitty A. The Anti-Mullerian Hormone Prodomain is Displaced From the Hormone/Prodomain Complex Upon Bivalent Binding to the Hormone Receptor. J Biol Chem (2022) 298:101429. doi: 10.1016/j.jbc.2021.101429 34801555 PMC8801479

[9] di Clemente N Wilson C Faure E Boussin L Carmillo P Tizard R. Cloning, Expression, and Alternative Splicing of the Receptor for Anti-Müllerian Hormone. Mol Endocrinol (1994) 8:1006–20. doi: 10.1210/mend.8.8.7997230 7997230

[10] di Clemente N Racine C Pierre A Taieb J. Anti-Müllerian Hormone in Female Reproduction. Endocr Rev (2021) 42:753–82. doi: 10.1210/endrev/bnab012 33851994

[11] Josso N. Women In Reproductive Science: Anti-Mullerian Hormone: A Look Back and Ahead. Reproduction (2019) 158:F81–9. doi: 10.1530/REP-18-0602 30844753

[12] Cohen-Haguenauer O Picard JY Mattei MG Serero S Nguyen VC de Tand MF. Mapping of the Gene for Anti-Mullerian Hormone to the Short Arm of Human Chromosome 19. Cytogenet Cell Genet (1987) 44:2–6. doi: 10.1159/000132332 3028714

[13] Guerrier D Boussin L Mader S Josso N Kahn A Picard JY. Expression of the Gene for Anti-Müllerian Hormone. JReprodFertil (1990) 88:695–706. doi: 10.1530/jrf.0.0880695 2325025

[14] Grinspon RP Rey RA. Anti-Mullerian Hormone and Sertoli Cell Function in Paediatric Male Hypogonadism. Horm Res Paediatr (2010) 73:81–92. doi: 10.1159/000277140 20190544

[15] Josso N Picard JY. Genetics of Anti-Mullerian Hormone and its Signaling Pathway. Best Pract Res Clin Endocrinol Metab (2022) 36:101634. doi: 10.1016/j.beem.2022.101634 35249805

[16] Lukas-Croisier C Lasala C Nicaud J Bedecarrás P Kumar TR Dutertre M. Follicle-Stimulating Hormone Increases Testicular Anti-Müllerian Hormone (AMH) Production Through Sertoli Cell Proliferation and a Nonclassical Cyclic Adenosine 5'-Monophosphate-Mediated Activation of the AMH Gene. Mol Endocrinol (2003) 17:550–61. doi: 10.1210/me.2002-0186 12554789

[17] Beau C Vivian N Münsterberg A Dresser DW Lovell-Badge R Guerrier D. *In Vivo* Analysis of the Regulation of the Anti-Müllerian Hormone, as a Marker of Sertoli Cell Differentiation During Testicular Development, Reveals a Multi-Step Process. Mol Reprod Dev (2001) 59:256–64. doi: 10.1002/mrd.1030 11424211

[18] Edelsztein NY Rey RA. Importance of the Androgen Receptor Signaling in Gene Transactivation and Transrepression for Pubertal Maturation of the Testis. Cells (2019) 8:1–17. doi: 10.3390/cells8080861 PMC6721648 31404977

[19] Valeri C Lovaisa MM Racine C Edelsztein NY Riggio M Giulianelli S. Molecular Mechanisms Underlying AMH Elevation in Hyperoestrogenic States in Males. Sci Rep (2020) 10:15062. doi: 10.1038/s41598-020-71675-7 32934281 PMC7492256

[20] Tran D Meusy-Dessolle N Josso N. Anti-Müllerian Hormone is a Functional Marker of Foetal Sertoli Cells. Nature (1977) 269:411–2. doi: 10.1038/269411a0 909589

[21] Münsterberg A Lovell-Badge R. Expression of the Mouse Anti-Müllerian Hormone Gene Suggests a Role in Both Male and Female Sexual Differentiation. Development (1991) 113:613–24. doi: 10.1242/dev.113.2.613 1782869

[22] Josso N Lamarre I Picard JY Berta P Davies N Morichon N. Anti-Müllerian Hormone in Early Human Development. Early Hum Dev (1993) 33:91–9. doi: 10.1016/0378-3782(93)90204-8 8055780

[23] Rey RA Musse M Venara M Chemes HE. Ontogeny of the Androgen Receptor Expression in the Fetal and Postnatal Testis: Its Relevance on Sertoli Cell Maturation and the Onset of Adult Spermatogenesis. Microscopy Res Technique (2009) 72:787–95. doi: 10.1002/jemt.20754 19551717

[24] Grinspon RP Bedecarrás P Ballerini MG Iñíguez G Rocha A Mantovani Rodrigues Resende EA. Early Onset of Primary Hypogonadism Revealed by Serum Anti-Müllerian Hormone Determination During Infancy and Childhood in Trisomy 21. Int J Androl (2011) 34:e487–98. doi: 10.1111/j.1365-2605.2011.01210.x 21831236

[25] Tran D Meusy-Dessolle N Josso N. Waning of Anti-Müllerian Activity: An Early Sign of Sertoli Cell Maturation in the Developing Pig. Biol Reprod (1981) 24:923–31. doi: 10.1095/biolreprod24.4.923 6894705

[26] Josso N Picard JY Dacheux JL Courot M. Detection of Anti-Müllerian Activity in Boar Rete Testis Fluid. J Reprod Fertil (1979) 57:397–400. doi: 10.1530/jrf.0.0570397 513029

[27] Rey R Lukas-Croisier C Lasala C Bedecarrás P. AMH/MIS: What We Know Already About the Gene, the Protein and Its Regulation. Mol Cell Endocrinology (2003) 211:21–31. doi: 10.1016/j.mce.2003.09.007 14656472

[28] Hirobe S He WW Lee MM Donahoe PK. Mullerian Inhibiting Substance Messenger Ribonucleic Acid Expression in Granulosa and Sertoli Cells Coincides With Their Mitotic Activity. Endocrinology (1992) 131:854–62. doi: 10.1210/endo.131.2.1639028 1639028

[29] Rey R Al-Attar L Louis F Jaubert F Barbet P Nihoul-Fékété C. Testicular Dysgenesis Does Not Affect Expression of Anti-Mullerian Hormone by Sertoli Cells in Premeiotic Seminiferous Tubules. Am J Pathology (1996) 148:1689–98. PMC1861550 8623936

[30] Al-Attar L Noël K Dutertre M Belville C Forest MG Burgoyne PS. Hormonal and Cellular Regulation of Sertoli Cell Anti-Müllerian Hormone Production in the Postnatal Mouse. J Clin Invest (1997) 100:1335–43. doi: 10.1172/JCI119653 PMC508311 9294098

[31] Hagen CP Aksglæde L Sorensen K Main KM Boas M Cleemann L. Serum Levels of Anti-Mullerian Hormone as a Marker of Ovarian Function in 926 Healthy Females From Birth to Adulthood and in 172 Turner Syndrome Patients. J Clin Endocrinol Metab (2010) 95:5003–10. doi: 10.1210/jc.2010-0930 20719830

[32] Jost A. Problems of Fetal Endocrinology: The Gonadal and Hypophyseal Hormones. Recent Prog Hormone Res (1953) 8:379–418.

[33] Grinspon RP Bergadá I Rey RA. Male Hypogonadism and Disorders of Sex Development. Front Endocrinol (Lausanne) (2020) 11:211. doi: 10.3389/fendo.2020.00211 32351452 PMC7174651

[34] Taguchi O Cunha GR Lawrence WD Robboy SJ. Timing and Irreversibility of Müllerian Duct Inhibition in the Embryonic Reproductive Tract of the Human Male. DevBiol (1984) 106:394–8. doi: 10.1016/0012-1606(84)90238-0 6548718

[35] Kuiri-Hänninen T Kallio S Seuri R Tyrvainen E Liakka A Tapanainen J. Postnatal Developmental Changes in the Pituitary-Ovarian Axis in Preterm and Term Infant Girls. J Clin Endocrinol Metab (2011) 96:3432–9. doi: 10.1210/jc.2011-1502 21900380

[36] Josso N Rey RA Picard JY. Anti-Müllerian Hormone: A Valuable Addition to the Toolbox of the Pediatric Endocrinologist. Int J Endocrinol (2013) 2013:674105. doi: 10.1155/2013/674105 24382961 PMC3870610

[37] Condorelli RA Cannarella R Calogero AE La Vignera S. Evaluation of Testicular Function in Prepubertal Children. Endocrine (2018) 62:274–80. doi: 10.1007/s12020-018-1670-9 29982874

[38] Rey RA. Biomarkers of Male Hypogonadism in Childhood and Adolescence. Adv Lab Med (2020) 2020:24. doi: 10.1515/almed-2020-0024 PMC10159267 37363780

[39] Kanakatti Shankar R Dowlut-McElroy T Dauber A Gomez-Lobo V. Clinical Utility of Anti-Mullerian Hormone in Pediatrics. J Clin Endocrinol Metab (2022) 107:309–23. doi: 10.1210/clinem/dgab687 PMC8764360 34537849

[40] Lee MM Donahoe PK Silverman BL Hasegawa T Hasegawa Y Gustafson ML. Measurements of Serum Müllerian Inhibiting Substance in the Evaluation of Children With Nonpalpable Gonads. New Engl J Med (1997) 336:1480–6. doi: 10.1056/NEJM199705223362102 9154766

[41] Josso N Rey RA. What Does AMH Tell Us in Pediatric Disorders of Sex Development? Front Endocrinol (Lausanne) (2020) 11:619. doi: 10.3389/fendo.2020.00619 33013698 PMC7506080

[42] Grinspon RP Rey RA. Molecular Characterization of XX Maleness. Int J Mol Sci (2019) 20:6089. doi: 10.3390/ijms20236089 PMC6928850 31816857

[43] Misra M MacLaughlin DT Donahoe PK Lee MM. Measurement of Mullerian Inhibiting Substance Facilitates Management of Boys With Microphallus and Cryptorchidism. J Clin Endocrinol Metab (2002) 87:3598–602. doi: 10.1210/jcem.87.8.8742 12161481

[44] Grinspon RP Gottlieb S Bedecarras P Rey RA. Anti-Müllerian Hormone and Testicular Function in Prepubertal Boys With Cryptorchidism. Front Endocrinol (Lausanne) (2018) 9:182:182. doi: 10.3389/fendo.2018.00182 29922225 PMC5996917

[45] Grinspon RP Castro S Brunello FG Sansó G Ropelato MG Rey RA. Diagnosis of Male Central Hypogonadism During Childhood. J Endocrine Society (2021) 5:1–8. doi: 10.1210/jendso/bvab145 PMC8475809 34589657

[46] Venara M Rey R Bergadá I Mendilaharzu H Campo SM Chemes H. Sertoli Cell Proliferations of the Infantile Testis: An Intratubular Form of Sertoli Cell Tumor? Am J Surg Pathol (2001) 25:1237–44. doi: 10.1097/00000478-200110000-00003 11688457

[47] Rey RA Venara M Coutant R Trabut JB Rouleau S Lahlou N. Unexpected Mosaicism of R201H-GNAS1 Mutant-Bearing Cells in the Testes Underlie Macro-Orchidism Without Sexual Precocity in McCune-Albright Syndrome. Hum Mol Genet (2006) 15:3538–43. doi: 10.1093/hmg/ddl430 17101633

[48] Rey R Lordereau-Richard I Carel JC Barbet P Cate RL Roger M. Anti-Müllerian Hormone and Testosterone Serum Levels Are Inversely Related During Normal and Precocious Pubertal Development. J Clin Endocrinol Metab (1993) 77:1220–6. doi: 10.1210/jc.2005-2564 8077315

[49] Bastida MG Rey RA Bergadá I Bedecarrás P Andreone L del Rey G. Establishment of Testicular Endocrine Function Impairment During Childhood and Puberty in Boys With Klinefelter Syndrome. Clin Endocrinol (Oxf) (2007) 67:863–70. doi: 10.1111/j.1365-2265.2007.02977.x 17645574

[50] Rey R Mebarki F Forest MG Mowszowicz I Cate RL Morel Y. Anti-Müllerian Hormone in Children With Androgen Insensitivity. J Clin Endocrinol Metab (1994) 79:960–4. doi: 10.1210/jcem.79.4.7962305 7962305

[51] Arango NA Lovell-Badge R Behringer RR. Targeted Mutagenesis of the Endogenous Mouse Mis Gene Promoter: *In Vivo* Definition of Genetic Pathways of Vertebrate Sexual Development. Cell (1999) 99:409–19. doi: 10.1016/s0092-8674(00)81527-5 10571183

[52] Shen WH Moore CC Ikeda Y Parker KL Ingraham HA. Nuclear Receptor Steroidogenic Factor 1 Regulates the Müllerian Inhibiting Substance Gene: A Link to the Sex Determination Cascade. Cell (1994) 77:651–61. doi: 10.1016/0092-8674(94)90050-7 8205615

[53] Schteingart HF Picard JY Valeri C Marshall I Treton D di Clemente N. A Mutation Inactivating the Distal SF1 Binding Site on the Human Anti-Mullerian Hormone Promoter Causes Persistent Mullerian Duct Syndrome. Hum Mol Genet (2019) 28:3211–8. doi: 10.1093/hmg/ddz147 31238341

[54] Viger RS Mertineit C Trasler JM Nemer M. Transcription Factor GATA-4 is Expressed in a Sexually Dimorphic Pattern During Mouse Gonadal Development and Is a Potent Activator of the Müllerian Inhibiting Substance Promoter. Development (1998) 125:2665–75. doi: 10.1242/dev.125.14.2665 9636081

[55] Nachtigal MW Hirokawa Y Enyeart-VanHouten DL Flanagan JN Hammer GD Ingraham HA. Wilms' Tumor 1 and Dax-1 Modulate the Orphan Nuclear Receptor SF-1 in Sex-Specific Gene Expression. Cell (1998) 93:445–54. doi: 10.1016/S0092-8674(00)81172-1 9590178

[56] Tremblay JJ Viger RS. Nuclear Receptor Dax-1 Represses the Transcriptional Cooperation Between GATA-4 and SF-1 in Sertoli Cells. Biol Reprod (2001) 64:1191–9. doi: 10.1095/biolreprod64.4.1191 11259267

[57] Bergadá I Milani C Bedecarrás P Andreone L Ropelato MG Gottlieb S. Time Course of the Serum Gonadotropin Surge, Inhibins, and Anti-Mullerian Hormone in Normal Newborn Males During the First Month of Life. J Clin Endocrinol Metab (2006) 91:4092–8. doi: 10.1210/jc.2006-1079 16849404

[58] Busch AS Ljubicic ML Upners EN Fischer MB Raket LL Frederiksen H. Dynamic Changes of Reproductive Hormones in Male Minipuberty: Temporal Dissociation of Leydig- and Sertoli-Cell Activity. J Clin Endocrinol Metab (2022). doi: 10.1210/clinem/dgac115 35225342

[59] Braslavsky D Grinspon RP Ballerini MG Bedecarrás P Loreti N Bastida G. Hypogonadotropic Hypogonadism in Infants With Congenital Hypopituitarism: A Challenge to Diagnose at an Early Stage. Horm Res Paediatr (2015) 84:289–97. doi: 10.1159/000439051 26355950

[60] Young J Chanson P Salenave S Noel M Brailly S O'Flaherty M. Testicular Anti-Mullerian Hormone Secretion Is Stimulated by Recombinant Human FSH in Patients With Congenital Hypogonadotropic Hypogonadism. J Clin Endocrinol Metab (2005) 90:724–8. doi: 10.1210/jc.2004-0542 15536161

[61] Bougnères P François M Pantalone L Rodrigue D Bouvattier C Demesteere E. Effects of an Early Postnatal Treatment of Hypogonadotropic Hypogonadism With a Continuous Subcutaneous Infusion of Recombinant Follicle-Stimulating Hormone and Luteinizing Hormone. J Clin Endocrinol Metab (2008) 93:2202–5. doi: 10.1210/jc.2008-0121 18381569

[62] Lasala C Schteingart HF Arouche N Bedecarrás P Grinspon RP Picard JY. SOX9 and SF1 are Involved in Cyclic AMP-Mediated Upregulation of Anti-Mullerian Gene Expression in the Testicular Prepubertal Sertoli Cell Line SMAT1. Am J Physiol Endocrinol Metab (2011) 301:E539-547. doi: 10.1152/ajpendo.00187.2011 21693691

[63] Mamkin I Philibert P Anhalt H Ten S Sultan C. Unusual Phenotypical Variations in a Boy With McCune-Albright Syndrome. Horm Res Paediatr (2010) 73:215–22. doi: 10.1159/000284365 20197676

[64] Berensztein EB Baquedano MS González CR Saraco NI Rodriguez J Ponzio R. Expression of Aromatase, Estrogen Receptor Alpha and Beta, Androgen Receptor, and Cytochrome P-450scc in the Human Early Prepubertal Testis. Pediatr Res (2006) 60:740–4. doi: 10.1203/01.pdr.0000246072.04663.bb 17065579

[65] Chemes HE Rey RA Nistal M Regadera J Musse M González-Peramato P. Physiological Androgen Insensitivity of the Fetal, Neonatal, and Early Infantile Testis Is Explained by the Ontogeny of the Androgen Receptor Expression in Sertoli Cells. J Clin Endocrinol Metab (2008) 93:4408–12. doi: 10.1210/jc.2008-0915 18713818

[66] Boukari K Meduri G Brailly-Tabard S Guibourdenche J Ciampi ML Massin N. Lack of Androgen Receptor Expression in Sertoli Cells Accounts for the Absence of Anti-Mullerian Hormone Repression During Early Human Testis Development. J Clin Endocrinol Metab (2009) 94:1818–25. doi: 10.1210/jc.2008-1909 PMC2699416 19276236

[67] Majdic G Millar MR Saunders PT. Immunolocalisation of Androgen Receptor to Interstitial Cells in Fetal Rat Testes and to Mesenchymal and Epithelial Cells of Associated Ducts. J Endocrinol (1995) 147:285–93. doi: 10.1677/joe.0.1470285 7490558

[68] Grinspon RP Andreone L Bedecarrás P Ropelato MG Rey RA Campo SM. Male Central Precocious Puberty: Serum Profile of Anti-Mullerian Hormone and Inhibin B Before, During, and After Treatment With GnRH Analogue. Int J Endocrinol (2013) 2013:823064. doi: 10.1155/2013/823064 24324495 PMC3845850

[69] Rey RA. The Role of Androgen Signaling in Male Sexual Development at Puberty. Endocrinology (2021) 162:bqaa215. doi: 10.1210/endocr/bqaa215 33211805

[70] Racine C Pask AJ Wijayanti GE di Clemente N Picard JY Shaw G. Early Expression of the Androgen Receptor in the Sertoli Cells of a Marsupial Coincides With Downregulation of Anti-Mullerian Hormone at the Time of Urogenital Virilization. Sex Dev (2009) 3:317–25. doi: 10.1159/000273263 20051675

[71] Almeida J Conley AJ Mathewson L Ball BA. Expression of Anti-Mullerian Hormone, Cyclin-Dependent Kinase Inhibitor (CDKN1B), Androgen Receptor, and Connexin 43 in Equine Testes During Puberty. Theriogenology (2012) 77:847–57. doi: 10.1016/j.theriogenology.2011.09.007 22115811

[72] Chang C Chen YT Yeh SD Xu Q Wang RS Guillou F. Infertility With Defective Spermatogenesis and Hypotestosteronemia in Male Mice Lacking the Androgen Receptor in Sertoli Cells. Proc Natl Acad Sci USA (2004) 101:6876–81. doi: 10.1073/pnas.0307306101 PMC406435 15107499

[73] Smith LB Walker WH. The Regulation of Spermatogenesis by Androgens. Semin Cell Dev Biol (2014) 30:2–13. doi: 10.1016/j.semcdb.2014.02.012 24598768 PMC4043871

[74] Denayer S Helsen C Thorrez L Haelens A Claessens F. The Rules of DNA Recognition by the Androgen Receptor. Mol Endocrinol (2010) 24:898–913. doi: 10.1210/me.2009-0310 20304998 PMC5417492

[75] Heckert LL Wilson EM Nilson JH. Transcriptional Repression of the {Alpha}-Subunit Gene by Androgen Receptor Occurs Independently of DNA Binding But Requires the DNA-Binding and Ligand-Binding Domains of the Receptor. Mol Endocrinol (1997) 11:1497–506. doi: 10.1210/mend.11.10.9996 PMC1502414 9280065

[76] Shang Y Myers M Brown M. Formation of the Androgen Receptor Transcription Complex. Mol Cell (2002) 9:601–10. doi: 10.1016/S1097-2765(02)00471-9 11931767

[77] Bulldan A Dietze R Shihan M Scheiner-Bobis G. Non-Classical Testosterone Signaling Mediated Through ZIP9 Stimulates Claudin Expression and Tight Junction Formation in Sertoli Cells. Cell Signal (2016) 28:1075–85. doi: 10.1016/j.cellsig.2016.04.015 27164415

[78] Gautam M Bhattacharya I Rai U Majumdar SS. Hormone Induced Differential Transcriptome Analysis of Sertoli Cells During Postnatal Maturation of Rat Testes. PloS One (2018) 13:e0191201. doi: 10.1371/journal.pone.0191201 29342173 PMC5771609

[79] Dutertre M Rey R Porteu A Josso N Picard JY. A Mouse Sertoli Cell Line Expressing Anti-Müllerian Hormone and Its Type II Receptor. Mol Cell Endocrinology (1997) 136:57–65. doi: 10.1016/s0303-7207(97)00214-1 9510068

[80] Edelsztein NY Racine C di Clemente N Schteingart HF Rey RA. Androgens Downregulate Anti-Mullerian Hormone Promoter Activity in the Sertoli Cell Through the Androgen Receptor and Intact SF1 Sites. Biol Reprod (2018) 99:1303–12. doi: 10.1093/biolre/ioy152 29985989

[81] Hero M Tommiska J Vaaralahti K Laitinen EM Sipila I Puhakka L. Circulating Antimullerian Hormone Levels in Boys Decline During Early Puberty and Correlate With Inhibin B. Fertil Steril (2012) 97:1242–7. doi: 10.1016/j.fertnstert.2012.02.020 22405597

[82] Grinspon R Chemes H Rey RA. Decline in Serum Antimullerian Hormone Due to Androgen Action in Early Puberty in Males. Fertil Steril (2012) 98:e23. doi: 10.1016/j.fertnstert.2012.06.003 22763099

[83] Rajpert-De Meyts E Jørgensen N Græm N Müller J Cate RL Skakkebæk NE. Expression of Anti-Müllerian Hormone During Normal and Pathological Gonadal Development: Association With Differentiation of Sertoli and Granulosa Cells. J Clin Endocrinol Metab (1999) 84:3836–44. doi: 10.1210/jcem.84.10.6047 10523039

[84] Rey RA Belville C Nihoul-Fékété C Michel-Calemard L Forest MG Lahlou N. Evaluation of Gonadal Function in 107 Intersex Patients by Means of Serum Antimüllerian Hormone Measurement. J Clin Endocrinol Metab (1999) 84:627–31. doi: 10.1210/jcem.84.2.5507 10022428

[85] Liu Q Yin X Li P. Clinical, Hormonal and Genetic Characteristics of Androgen Insensitivity Syndrome in 39 Chinese Patients. Reprod Biol Endocrinol (2020) 18:34. doi: 10.1186/s12958-020-00593-0 32345305 PMC7187512

[86] Lan KC Chen YT Chang C Chang YC Lin HJ Huang KE. Up-Regulation of SOX9 in Sertoli Cells From Testiculopathic Patients Accounts for Increasing Anti-Mullerian Hormone Expression *via* Impaired Androgen Receptor Signaling. PloS One (2013) 8:e76303. doi: 10.1371/journal.pone.0076303 24098470 PMC3788123

[87] Dorrington JH Armstrong DT. Follicle-Stimulating Hormone Stimulates Estradiol-17beta Synthesis in Cultured Sertoli Cells. Proc Natl Acad Sci USA (1975) 72:2677–81. doi: 10.1073/pnas.72.7.2677 PMC432833 170613

[88] Nilsson S Makela S Treuter E Tujague M Thomsen J Andersson G. Mechanisms of Estrogen Action. Physiol Rev (2001) 81:1535–65. doi: 10.1152/physrev.2001.81.4.1535 11581496

[89] Prossnitz ER Barton M. Estrogen Biology: New Insights Into GPER Function and Clinical Opportunities. Mol Cell Endocrinol (2014) 389:71–83. doi: 10.1016/j.mce.2014.02.002 24530924 PMC4040308

[90] Cooke PS Nanjappa MK Ko C Prins GS Hess RA. Estrogens in Male Physiology. Physiol Rev (2017) 97:995–1043. doi: 10.1152/physrev.00018.2016 28539434 PMC6151497

[91] Lucas TFG Lazari MFM Porto CS. Differential Role of the Estrogen Receptors ESR1 and ESR2 on the Regulation of Proteins Involved With Proliferation and Differentiation of Sertoli Cells From 15-Day-Old Rats. Mol Cell Endocrinol (2014) 382:84–96. doi: 10.1016/j.mce.2013.09.015 24056172

[92] Lucas TF Royer C Siu ER Lazari MF Porto CS. Expression and Signaling of G Protein-Coupled Estrogen Receptor 1 (GPER) in Rat Sertoli Cells. Biol Reprod (2010) 83:307–17. doi: 10.1095/biolreprod.110.084160 20445128

[93] Grinspon RP Urrutia M. The Importance of Follicle-Stimulating Hormone in the Prepubertal and Pubertal Testis. Curr Opin Endocrine Metab Res (2020) 14:137–44. doi: 10.1016/j.coemr.2020.07.007

[94] Grynberg M Pierre A Rey R Leclerc A Arouche N Hesters L. Differential Regulation of Ovarian Anti-Mullerian Hormone (AMH) by Estradiol Through Alpha- and Beta-Estrogen Receptors. J Clin Endocrinol Metab (2012) 97:E1649-1657. doi: 10.1210/jc.2011-3133 22689696

[95] Yang WR Zhu FW Zhang JJ Wang Y Zhang JH Lu C. PI3K/Akt Activated by GPR30 and Src Regulates 17beta-Estradiol-Induced Cultured Immature Boar Sertoli Cells Proliferation. Reprod Sci (2017) 24:57–66. doi: 10.1177/1933719116649696 27222231

[96] Matthiesson KL Stanton PG O'Donnell L Meachem SJ Amory JK Berger R. Effects of Testosterone and Levonorgestrel Combined With a 5alpha-Reductase Inhibitor or Gonadotropin-Releasing Hormone Antagonist on Spermatogenesis and Intratesticular Steroid Levels in Normal Men. J Clin Endocrinol Metab (2005) 90:5647–55. doi: 10.1210/jc.2005-0639 16030154

[97] Bernard V Kherra S Francou B Fagart J Viengchareun S Guechot J. Familial Multiplicity of Estrogen Insensitivity Associated With a Loss-Of-Function ESR1 Mutation. J Clin Endocrinol Metab (2017) 102:93–9. doi: 10.1210/jc.2016-2749 PMC5413105 27754803

